# Integrative Analysis of Transcriptomic and Metabolomic Profiles Uncovers the Mechanism of Color Variation in the Tea Plant Callus

**DOI:** 10.3390/plants14101454

**Published:** 2025-05-13

**Authors:** Mengna Xiao, Yingju Tian, Ya Wang, Yunfang Guan, Ying Zhang, Yuan Zhang, Yanlan Tao, Zengquan Lan, Dexin Wang

**Affiliations:** 1 College of Landscape Architecture and Horticulture, Southwest Forestry University, Kunming 650224, China; 1xiao@swfu.edu.cn (M.X.); 2517733709@swfu.edu.cn (Y.T.); 7_ww@swfu.edu.cn (Y.W.); 2 College of Forestry, Southwest Forestry University, Kunming 650224, China; g_yunfang@swfu.edu.cn (Y.G.); zhangying@swfu.edu.cn (Y.Z.); yuanzhang@swfu.edu.cn (Y.Z.); 317924340@swfu.edu.cn (Y.T.); lzq@swfu.edu.cn (Z.L.); 3 Engineering Research Center for the Development and Utilization of Forest Resources in the Field of Big Health in Yunnan Provincial Universities, Kunming 650224, China; 4 Ancient Tea Tree Research Center of Southwest Forestry University, Kunming 650224, China; 5 Graduate School of Southwest Forestry University, Kunming 650224, China

**Keywords:** *Camellia sinensis*, callus, transcriptome, metabolome, flavonoid, photosynthesis

## Abstract

Tea plants (*Camellia sinensis*) are among the world’s most significant economic tree species. Tissue culture serves as a crucial method in commercial breeding by facilitating the rapid propagation of valuable genotypes and the generation of disease-free clones. However, callus browning represents a prevalent challenge in tea plant tissue culture, and may adversely affect explant growth and development. Our research demonstrates that although anti-browning agents can effectively suppress browning, they induce distinct color changes in the callus. These color variations could significantly influence callus induction and subsequent growth patterns. In this study, callus tissues from *C. sinensis* var. *Assamica* cv. Mengku were employed as experimental materials and treated with three commonly used anti-browning agents: ascorbic acid (VC), activated carbon (AC), and polyvinylpyrrolidone (PVP). The results demonstrated that while these three reagents effectively inhibited browning, they also induced distinct color changes in the explants, which appeared red, green, and white, respectively. Furthermore, this study investigated the molecular mechanisms underlying callus color changes using transcriptomic and metabolomic approaches. Based on transcriptome analysis, it was revealed that photosynthesis and flavonoid biosynthesis pathways were significantly enriched. Metabolome analysis identified 14 phenolic acids, which exhibited significant variation in accumulation across calluses of different colors. The differential expression of genes involved in flavonoid biosynthesis pathways, coupled with the distinct accumulation patterns of metabolites, can effectively alleviate photooxidative damage and enhance the resistance of callus to browning. AC activates the photosynthesis of callus by regulating carbon source allocation and upregulating the expression of key genes in the *psa*, *psb*, and *pet* families within the photosynthetic system. This process promotes chlorophyll biosynthesis, thereby enabling the callus to grow green, while VC activates the expression of key genes such as *CHS*, *F3H*, *C4H*, *CYP75B1*, and *ANR* in the flavonoid pathway, which are involved in the regulation of pigment synthesis in red callus. This study elucidated the molecular mechanisms underlying the effects of anti-browning agents on color variations in *C. sinensis* callus, thereby providing a robust theoretical foundation for optimization, the establishment of tea plant tissue culture systems, and enhancing cultivar quality.

## 1. Introduction

*C. sinensis* (L.) O. Kuntze, belonging to the genus *Camellia* (family Theaceae), is a perennial evergreen woody plant. It is widely cultivated in provinces such as Fujian, Guangdong, Yunnan, Guizhou, and Sichuan in China, with southwestern China being recognized as the origin of the world’s tea plants [1]. Tea has achieved global popularity owing to its unique sensory characteristics and health-benefiting properties, establishing itself as one of the three major non-alcoholic beverages worldwide and a crucial agricultural commodity [2]. Fresh tea leaves are rich in a diverse array of metabolites, including amino acids, flavonoids, and phenolic acids [3,4,5,6]. These bioactive compounds play a crucial role in conferring tea’s multiple health benefits, such as antioxidation, anti-aging effects, anticancer properties, and hypotensive activities. Consequently, tea is extensively processed in a wide range of food and pharmaceutical products [7].

As a crucial economic crop, the large-scale propagation and quality enhancement of tea plants remain central challenges in the tea industry. With the advent of modern biotechnology, tissue culture technology has become a pivotal method for the rapid propagation of elite tea germplasms. However, during tissue culture, callus browning occurs frequently, which not only impairs callus growth and differentiation, but also significantly hinders the establishment of efficient rapid propagation systems for elite cultivars of tea plants [8]. Browning refers to the process of pigment degradation or aggregation, which can be caused by either enzymatic or non-enzymatic mechanisms [9]. Among these, enzymatic browning is the primary factor contributing to browning in plant callus. This phenomenon occurs when phenolic compounds interact with polyphenol oxidase in explants or callus, producing quinones through oxidation reactions. In contrast, non-enzymatic browning is primarily induced by adverse external conditions, resulting in cell death and subsequent browning [10,11,12]. Through the synergistic application of the phenylalanine ammonia lyase (*PAL*) specific inhibitor 2-aminoindane-2-phosphonic acid (AIP; 2 μM) and systematic optimization of the tissue culture system, oxidative browning in tea plant tissue culture was effectively mitigated [13]. Additionally, in studies aimed at controlling explant browning during *C. sinensis* tissue culture, the medium composition was refined by incorporating antioxidants and adsorbents [11]. In conclusion, the browning phenomenon is one of the most significant limiting factors in establishing *C. sinensis* callus culture systems. Further investigation into effective anti-browning strategies could improve the success rate and efficiency of tea plant tissue culture. At present, the application of anti-browning agents in anti-browning measures has important practical value due to its simple operation and remarkable effect. Common anti-browning agents include citric acid, ascorbic acid, polyvinylpyrrolidone, activated carbon, and tea polyphenols. Among them, activated carbon reduces browning and maintains the lighter color of the callus by adsorbing phenolic substances and their oxidation products, thereby inhibiting oxidation reactions [14]. As a potent reducing agent, ascorbic acid converts quinones back into phenols while consuming oxygen, effectively delaying or preventing the callus from turning dark brown [15]. Tea polyphenols mitigate oxidative damage by scavenging reactive oxygen species (ROS) and interrupting free radical chain reactions, thus preserving the callus’ healthy colors, such as light green or white, and preventing browning discoloration [16].

Color, serving as a critical indicator of the health status and developmental stage of callus, is frequently closely associated with intracellular pigment synthesis, the activity of the antioxidant system, and the expression patterns of specific genes [17]. Under normal conditions, the biosynthesis and degradation of chlorophyll in plants maintain a dynamic equilibrium. However, any alteration in the expression levels of genes associated with the chlorophyll degradation pathway can disrupt this balance, resulting in abnormal pigmentation in plants [18]. A substantial body of research has consistently shown that carotenoids, flavonoids, and alkaloids are critical to the formation of plant color [19]. Specifically, the concentration and composition of chlorophyll, flavonoids, and carotenoids have a direct impact on color development in tea plants [20]. Chloroplasts, serving as the primary sites of photosynthesis, contain chlorophyll that directly determines the development of plant green phenotypes. Under dark conditions, callus cells generally fail to differentiate into chloroplasts, leading to chlorophyll deficiency and a resultant white, cream-colored, or translucent appearance [21]. Carotenoids, as lipid-soluble photosynthetic pigments that are ubiquitously present in the plant, exhibit chemical diversity in their molecular structures, which determines the characteristic color phenotypes ranging from yellow to orange and red in plant tissues.

Flavonoids, recognized as one of the most significant groups of secondary metabolites in plants [22], encompass anthocyanins, catechins, flavones, and flavonols, serving as major pigments responsible for red, pink, and purple–blue phenotypes in plants [23]. The biosynthesis of flavonoids begins with the phenylpropanoid pathway [24], in which L-phenylalanine acts as the precursor and undergoes sequential catalysis via phenylalanine ammonia lyase (*PAL*), cinnamate 4-hydroxylase (*C4H*), and 4-coumarate-CoA ligase enzymes (*4CL*) to produce 4-coumaroyl-CoA [25]. This intermediate subsequently reacts with malonyl-CoA under the catalytic action of chalcone synthase (*CHS*) and stilbene synthase (*STS*), leading to the formation of chalcone and resveratrol, respectively. Chalcone then undergoes a series of enzymatic modifications mediated by *CHI* (chalcone isomerase), *F3H* (flavanone 3-hydroxylase), and *F3′H* (flavonoid 3′-hydroxylase), culminating in the generation of structurally diverse flavonoid compounds [24]. Studies have demonstrated that yellow to albino tea leaves exhibit chloroplast defects and reduced levels of chlorophyll and carotenoids, as well as inhibited flavonoid biosynthesis [26,27]. The red or purple phenotypes of tea leaves are attributed to elevated anthocyanin accumulation and decreased chlorophyll content [28], which highlights the pivotal role of the altered dynamic equilibrium of secondary metabolites in plant coloration processes.

While the molecular mechanisms underlying plant coloration have been extensively studied, research on *C. sinensis* has primarily focused on leaf coloration, with significantly less attention given to the secondary metabolic processes associated with callus color changes. This study screened three typical anti-browning agents—ascorbic acid (VC), activated carbon (AC), and polyvinylpyrrolidone (PVP)—via preliminary experiments. The results demonstrated that these agents not only effectively inhibited browning progression, but also induced distinct phenotypic differentiation. Specifically, callus treated with VC exhibited red coloration, AC-treated callus appeared to be green, and PVP-treated callus remained white. This color heterogeneity indicates that various anti-browning agents may influence the accumulation of color compounds by specifically modulating gene expression and metabolic pathways. To investigate the molecular mechanisms underlying color changes in *C. sinensis* callus, this study combined a comparative analysis of color trait differences under different anti-browning agent treatments with transcriptomic and metabolomic profiling to explore the metabolic expression patterns and gene regulatory mechanisms involved in callus discoloration. The research findings can enhance our understanding of browning phenomena and their control mechanisms in *C. sinensis* tissue culture, offering a solid scientific foundation and reliable technical support for the rapid propagation and genetic improvement of elite tea cultivars.

## 2. Results

### 2.1. Analysis of Differentially Expressed Genes (DEGs)

**Figure 1:**
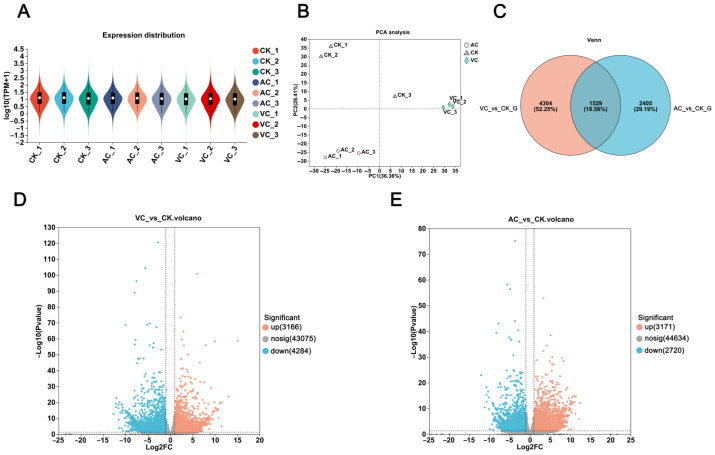
Comprehensive transcriptomic analysis of *C. sinensis* callus. (**A**) Violin plot illustrating the distribution of DEGs in callus. (**B**) Principal Component Analysis (PCA) plot demonstrating the variation among callus samples. (**C**) Venn diagram depicting the number of overlapping DEGs across different groups. (**D**) Volcano plot showing DEGs identified in the VC vs. CK comparison. (**E**) Volcano plot highlighting DEGs detected in the AC vs. CK comparison.

To explore the molecular mechanisms underlying color changes in callus induced by different anti-browning agents and to elucidate the associated gene expression patterns, transcriptome sequencing was conducted on callus samples from three groups: the AC treatment group (green), the VC treatment group (red), and the CK control group (white). Each group included three biological replicates to ensure the reliability of the data (Table S1). The results demonstrated that the AC treatment group (green) exhibited a higher median gene expression with a more concentrated distribution density. In contrast, the VC treatment group (red) showed a broader distribution range and a higher degree of dispersion. The CK control group (white), on the other hand, presented an intermediate distribution pattern; however, its overall expression level was significantly lower than that of both the AC and VC treatment groups (Figure 1A). Principal Component Analysis (PCA) revealed distinct clustering patterns among samples treated with AC, VC, and CK (Figure 1B). These findings suggest that the transcriptomic data are highly reproducible and reliable, thereby confirming their appropriateness for subsequent in-depth analysis. The Venn diagram analysis demonstrated that 4304 genes were exclusively expressed in VC and CK, while 2405 genes were uniquely expressed in AC and CK. Notably, the overlap of 1529 genes highlighted substantial interactions among calluses of different colors (Figure 1C). In the comparisons of VC vs. CK and AC vs. CK, 7450 and 5891 significant DEGs were identified, respectively. Among these, 3166 and 3171 genes were upregulated, while 4284 and 2720 genes were downregulated in each comparison (Figure 1D,E).

### 2.2. Enrichment Analysis

**Figure 2:**
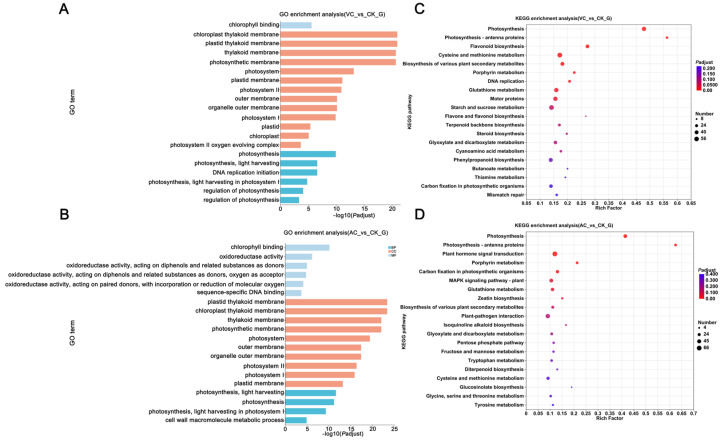
KEGG and GO enrichment analysis. (**A**) GO enrichment analysis of DEGs in VC vs. CK; (**B**) GO enrichment analysis of DEGs in AC vs. CK; (**C**) KEGG enrichment analysis of DEGs in VC vs. CK; and (**D**) KEGG enrichment analysis of DEGs in AC vs. CK. BP: Biological Process; CC: Cellular Component; and MF: Molecular Function. The size of the dot represents the number of genes involved in the pathway, while the color gradient reflects the adjusted *p*-value, transitioning from blue (less significant, higher *p*-value) to red (more significant, lower *p*-value).

To characterize the transcriptional expression patterns of differently colored calluses, we conducted the functional annotation of DEGs using the Gene Ontology (GO) database. The GO enrichment analysis revealed that DEGs in both the VC vs. CK and AC vs. CK comparison groups were predominantly enriched in pathways related to photosynthesis (Figure 2A,B). In the Biological Process (BP) category, DEGs associated with photosynthesis were significantly more enriched in the VC vs. CK comparison than in other components. By contrast, DEGs involved in photosynthesis light harvesting exhibited higher enrichment levels in the AC vs. CK comparison relative to other categories. In the Cellular Component (CC) category, both comparisons (VC vs. CK and AC vs. CK) demonstrated the significant enrichment of genes related to the thylakoid membrane system. In the Molecular Function (MF) category, DEGs in the VC vs. CK comparison were specifically enriched in chlorophyll binding, while those in the AC vs. CK comparison were associated with chlorophyll binding, oxidoreductase activity, and DNA binding.

Further enrichment analysis was conducted using the Kyoto Encyclopedia of Genes and Genomes (KEGGs) database to identify the metabolic pathways associated with significant DEGs. The top 20 enriched pathways in both comparison groups were visualized for clearer interpretation. In the VC vs. CK comparison, DEGs were significantly enriched in pathways related to photosynthesis (including antenna proteins), flavonoid biosynthesis, and cysteine and methionine metabolism, as well as the biosynthesis of various plant secondary metabolites. In the AC vs. CK comparison, DEGs were significantly enriched in pathways associated with photosynthesis (including antenna proteins), plant hormone signal transduction, porphyrin metabolism, and carbon fixation in photosynthetic organisms. Combined GO and KEGG results suggest that photosynthesis and flavonoid biosynthesis pathways may be key in regulating callus color change (Figure 2C,D). Combined GO and KEGG enrichment analysis results indicate that the photosynthesis and flavonoid biosynthesis pathways may play crucial roles in regulating callus color changes (Figure 2C,D).

### 2.3. Analysis of DEGs Related to Photosynthesis-Related Synthetic Pathways

**Figure 3:**
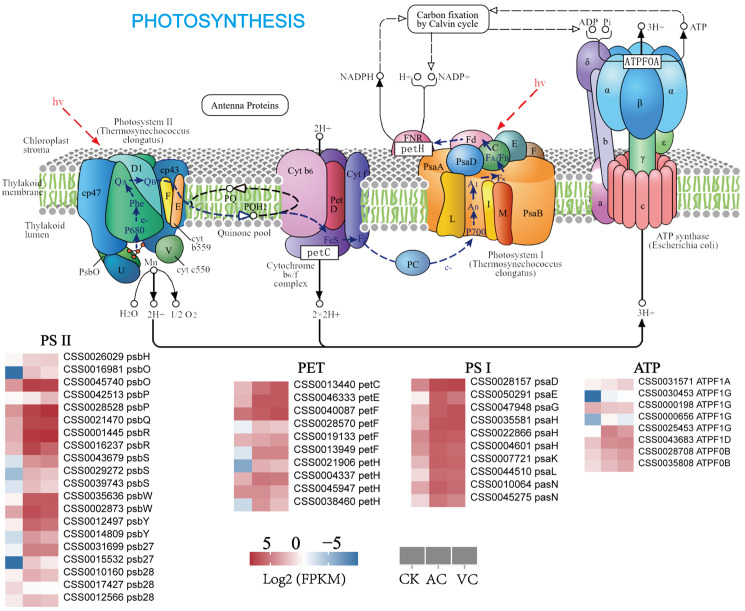
Expression patterns of photosynthesis-related genes in *C. sinensis* callus. The heatmap illustrates gene expression levels across three treatment groups (VC, AC, and CK), with the color gradient transitioning from blue (low expression) to red (high expression). This pathway is derived from the KEGG photosynthesis pathway.

Photosynthesis is a critical process for pigment biosynthesis in plants, particularly for chlorophyll, the primary photosynthetic pigment, whose biosynthesis is closely linked to the development of the photosynthetic system [29]. In this pathway, 48 photosynthesis-related DEGs showed significant expression pattern variations under the three treatment conditions (Figure 3 and Table S2). In the VC treatment group, 45 DEGs were primarily categorized into four major functional modules: 18 genes from the psb family involved in Photosystem II (PS II) function, 10 genes from the psa family associated with Photosystem I (PS I), 10 genes related to the cytochrome b6/f complex and photosynthetic electron transport, and 7 genes belonging to the F-type ATPase complex. In the AC treatment group, 39 DEGs were primarily categorized into four major functional modules: 17 genes from the psb family involved in Photosystem II (PS II) function, 10 genes from the psa family associated with Photosystem I (PSI), 7 genes related to the cytochrome b6/f complex and photosynthetic electron transport, and 5 genes belonging to the F-type ATPase complex. However, these genes showed a relatively low expression pattern in the CK control group.

### 2.4. Relative Expression Analysis of Selected Genes in Photosynthesis Process

**Figure 4:**
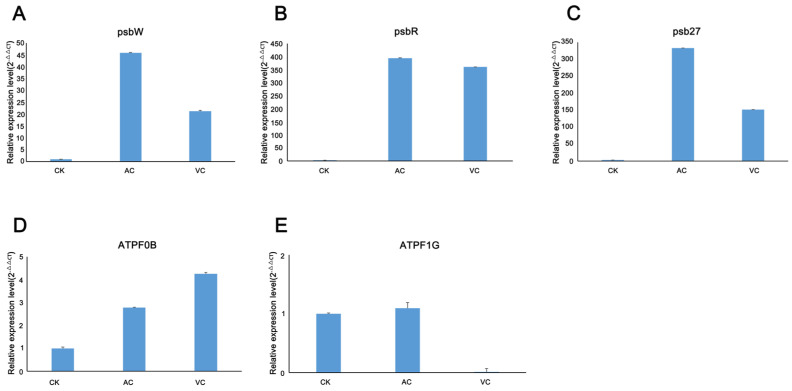
Quantitative real-time PCR analysis of differentially expressed DEGs involved in the photosynthesis pathway. (**A**–**E**) psbW, psbR, psb27, ATPF0B and ATPF1G represent the relative expression levels in callus treated with CK, AC and VC, respectively.

Given that the addition of anti-browning agents influences the growth and development of *C. sinensis* callus by regulating photosynthesis, the transcript levels of these genes significantly upregulated the expression of AC and VC samples compared to CK. We employed quantitative real-time PCR (qRT-PCR) to validate the expression levels of the selected genes. Specifically, five genes from the photosynthesis pathway were chosen for validation (Table S3). The results demonstrated that their expression patterns aligned well with the trends observed in the transcriptomic data (Figure 4).

### 2.5. Metabolite Profiling of Callus Tissues with Different Colors

**Figure 5:**
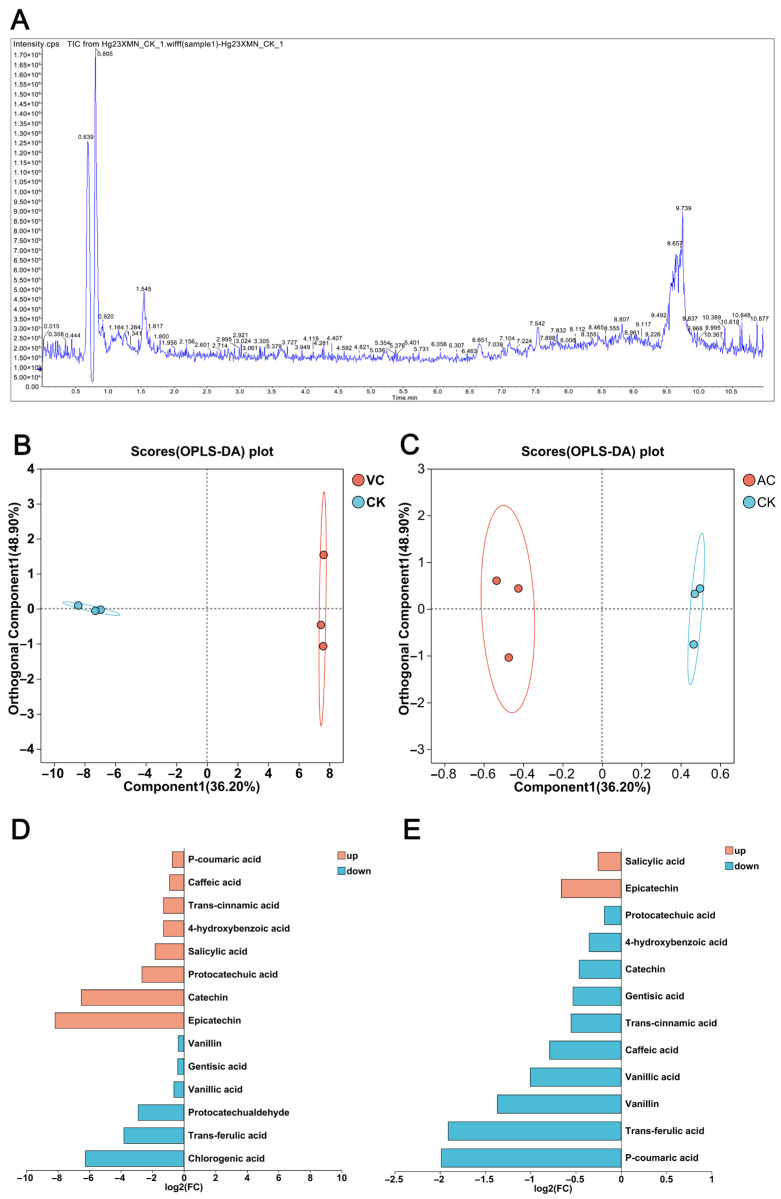
Metabolome analysis of *C. sinensis* callus. (**A**) Total ion chromatogram of callus; (**B**) PCA plot of metabolites in VC vs. CK; (**C**) PCA plot of metabolites in AC vs. CK; (**D**) fold–change bar plot of VC treatment (red callus); and (**E**) fold–change bar plot of AC treatment (green callus).

To elucidate the dynamic changes in metabolite profiles during the color variation in different callus types, this study utilized a targeted metabolomics approach based on liquid chromatography–mass spectrometry (LC-MS) to analyze the phenolic acid metabolic profiles of calluses with different colors. A total of 14 phenolic acid compounds were identified, and their relative abundances were visualized through the total ion chromatogram (TIC) (Figure 5A). The stable ion current signals confirmed the reliable performance of the mass spectrometry detection system. To ensure the reliability of the methods and data, Principal Component Analysis (PCA) was conducted on all compounds detected via liquid chromatography–mass spectrometry (LC-MS). The results demonstrated that the three biological replicates within each group were tightly clustered, while distinct separation was observed between groups (Figure 5B,C). This indicates substantial metabolic differences among the callus samples of different colors and confirms the robustness and reliability of the data for subsequent analysis. Analysis of the fold–change bar charts revealed the changes in metabolites between VC vs. CK and AC vs. CK. In the comparison group of VC vs. CK, a total of 14 differential phenolic acid metabolites were identified. Among them, eight metabolites showed significantly upregulated expression, and six metabolites showed a downward trend. In the comparison group of AC vs. CK, 12 differentially expressed metabolites (DEMs) were detected, including 2 upregulated metabolites and 10 downregulated metabolites (Figure 5D,E).

### 2.6. Enrichment Analysis of Differential Metabolites (DEMs)

**Figure 6:**
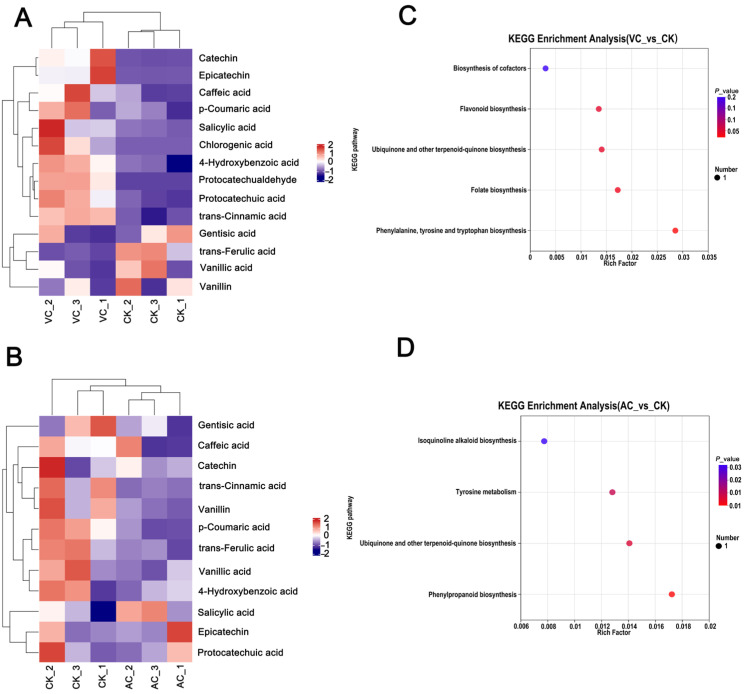
Differential accumulation of metabolites in *C. sinensis* callus. Differential metabolites (DEMs) were defined as those showing significant variations in abundance (adjusted *p* < 0.05, fold change > 1) between treatment groups (VC vs. CK, AC vs. CK) based on LC-MS data analysis. (**A**) Heatmap of metabolites in red callus (VC). (**B**) Heatmap of metabolites in green callus (AC). The *x*-axis denotes different experimental groups, while the *y*-axis indicates various metabolites. The color blocks at each position reflect the relative expression levels of metabolites between the two groups, with red indicating high expression and purple indicating low expression. (**C**) KEGG pathway enrichment analysis of metabolites in VC vs. CK; (**D**) KEGG pathway enrichment analysis of metabolites in AC vs. CK.

Among the identified metabolites, phenolic acids were detected in different calluses. The results demonstrated significant differences in the expression profiles of phenolic acid metabolites across various groups. In the VC vs. CK comparison, the relative expression levels of 4-hydroxybenzoic acid, epicatechin, catechin, protocatechuic acid, and trans-cinnamic acid were significantly upregulated, whereas those of trans-ferulic acid and vanillic acid were significantly downregulated (Figure 6A). In the AC vs. CK comparison, salicylic acid was upregulated in the AC treatment, while most other phenolic acid metabolites, such as *p*-coumaric acid and trans-cinnamic acid, were significantly downregulated (Figure 6B).

To further elucidate the metabolic regulatory mechanisms underlying callus color changes under different treatments, KEGG enrichment analysis was performed on the DEMs identified across multiple comparison groups. In the VC vs. CK group, metabolic pathways with significant enrichment were primarily categorized into five groups: biosynthesis of cofactors, flavonoid biosynthesis, ubiquinone and other terpenoid–quinone biosynthesis, folate biosynthesis, phenylalanine, tyrosine, and tryptophan biosynthesis (Figure 6C). In the AC vs. CK group, metabolic pathways with significant enrichment were primarily categorized into four groups: isoquinoline alkaloid biosynthesis, tyrosine metabolism, ubiquinone and other terpenoid–quinone biosynthesis, and phenylpropanoid biosynthesis (Figure 6D). Notably, when integrated with transcriptomic KEGG enrichment analysis, the “flavonoid biosynthesis” pathway was found to be enriched in both analyses. This suggests that this pathway may play a pivotal role in the color changes in *C. sinensis* callus.

### 2.7. Integrated Analysis of Transcriptome and Metabolome

**Figure 7:**
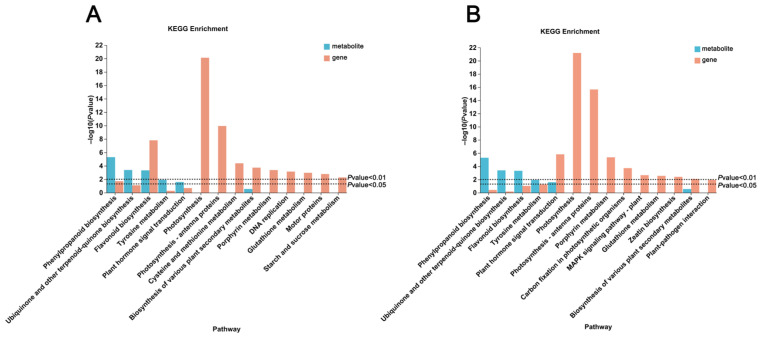
KEGG enrichment analysis results of DEGs and DEMs. (**A**) Combined KEGG enrichment analysis results for DEGs and DEMs in the comparison of VC vs. CK. (**B**) Combined KEGG enrichment analysis results for DEGs and DEMs in the comparison of AC vs. CK.

To gain a more comprehensive understanding of the metabolic expression and gene regulation associated with color changes in *C. sinensis* callus treated with different anti-browning agents, an integrated metabolomics and transcriptomics analysis was performed. DEGs and metabolites from the treatment groups were functionally characterized through annotation against the KEGG database (Figure 7). The results revealed that the KEGG pathways enriched with DEGs and metabolites showed a high degree of similarity between the VC vs. CK and AC vs. CK groups. These pathways were primarily associated with phenylpropanoid biosynthesis, ubiquinone and other terpenoid–quinone biosynthesis, flavonoid biosynthesis, tyrosine metabolism, plant hormone signal transduction, and the biosynthesis of secondary metabolites in plants. Analysis of the enriched metabolic pathways revealed a significant correlation between differentially expressed genes and metabolites in the flavonoid biosynthesis pathway and callus color.

### 2.8. Flavonoid Biosynthesis Pathway

**Figure 8:**
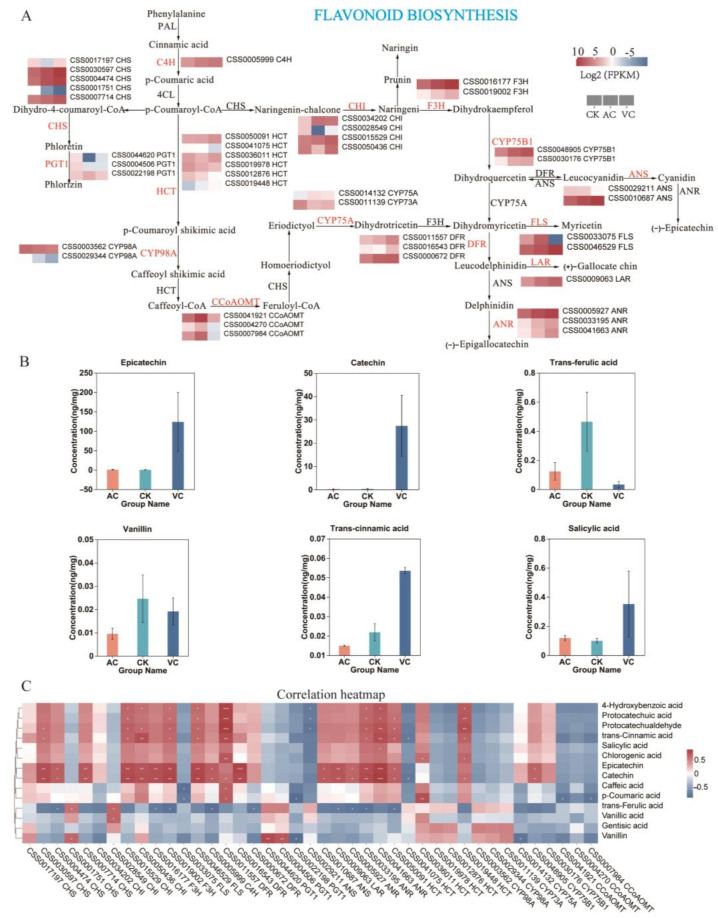
Transcriptomic and metabolomic changes in genes involved in the flavonoid biosynthesis pathway. (**A**) Genes participating in flavonoid biosynthesis. (**B**) Key metabolites involved in flavonoid metabolism. (**C**) Correlation analysis between differentially expressed genes and metabolites in the flavonoid biosynthesis pathway. CK: control group; VC: red callus; and AC: green callus. In the heatmap, red indicates high expression, and blue indicates low expression. Asterisks indicate significance (* *p* < 0.05, ** *p* < 0.01, *** *p* < 0.001).

The transcriptional abundance of 41 DEGs involved in the flavonoid biosynthesis pathway was analyzed using transcriptomic data (Figure 8A and Table S4). Among these, the 3 chalcone synthase (CHS), 2 chalcone isomerase (CHI), 2 flavanone 3-hydroxylase (F3H), 3 Dihydroflavonol 4-reductase (DFR), 1 Anthocyanidin synthase (ANS), 1 Leucoanthocyanidin Reductase (LAR), 1 Cinnamate-4-Hydroxylase (C4H), 3 Anthocyanidin reductase (ANR), 1 flavonol synthase (FLS), and 2 flavonoid 3′-monooxygenase (CYP75B1) genes were significantly upregulated in VC (Figure 8A).

Notably, in the anti-browning agent-treated groups, several key metabolites involved in the flavonoid biosynthetic pathway were identified, including epicatechin, catechin, trans-ferulic acid, trans-cinnamic acid, vanillic acid, and salicylic acid. These six metabolites showed significant variations in their expression levels across different treatment groups. Among these, four metabolites—epicatechin, catechin, trans-cinnamic acid, and salicylic acid—were found to exhibit significantly higher levels in red callus compared with white and green callus. Conversely, trans-ferulic acid and vanillic acid showed significantly greater accumulation in white callus than in red and green callus. Notably, the level of salicylic acid was observed to be higher in green callus than in white callus (Figure 8B). Correlation analyses of DEMs and DEGs revealed that six metabolites—including epicatechin, catechin, trans-ferulic acid, trans-cinnamic acid, vanillic, and salicylic acid—were significantly correlated with genes in the flavonoid biosynthesis pathway (Figure 8C). Among these, three metabolites—epicatechin, catechin, and trans-cinnamic acid—were significantly positively correlated with key enzymes in the flavonoid biosynthesis pathway, including *ANR*, *FLS*, *F3H*, *CHS*, *CHI*, *CYP75B1*, and *C4H*. Conversely, they were negatively correlated with genes such as *CCoAOMT*, *HCT*, and *PGT1*. And trans-ferulic acid and vanillic acid showed predominantly negative correlations with most genes in this pathway (Figure 8C).

### 2.9. Validation of Relative Expression of Selected Genes in the Flavonoid Biosynthesis Pathway via qRT-PCR

**Figure 9:**
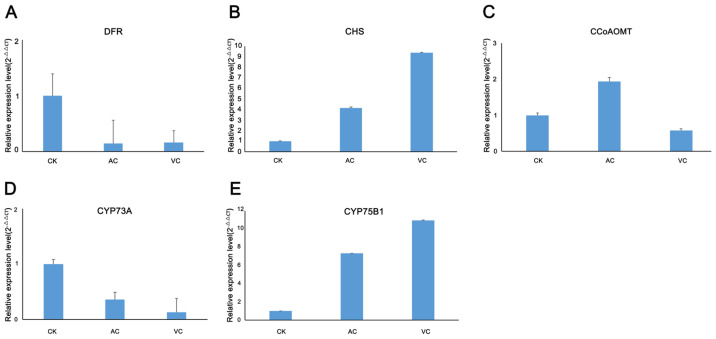
qRT-PCR analysis of DEGs in the flavonoid biosynthesis pathway. (**A**–**E**) DFR, CHS, CCoAOMT, CYP73A and CYP75B1represent the relative expression levels in callus treated with CK, AC and VC, respectively.

Given that the addition of anti-browning agents influences pigment synthesis in callus by regulating the flavonoid biosynthesis pathway, we validated the expression levels of selected genes using qRT-PCR. Specifically, five genes from the flavonoid biosynthesis pathway were chosen for validation. The results demonstrated that their expression patterns were consistent with the trends observed in the transcriptomic data (Figure 9).

## 3. Discussion

The browning of plant callus is a prevalent challenge in tissue culture that significantly compromises growth and regeneration capabilities, ultimately diminishing the efficiency of plant propagation [8]. In *C. sinensis*, the abundance of polyphenolic compounds leads to their oxidation into brown quinones at cut surfaces. This process not only induces toxicity in explants, but also exacerbates browning and contributes to tissue necrosis [30]. Although plants enhance the biosynthesis of phenolic compounds to withstand harsh environments, excessive accumulation of these compounds in callus tissue can lead to increased quinone production, ultimately resulting in severe browning [31]. During callus induction, browning not only hinders the growth and differentiation of explants, but may also decrease the success rates of induction. Current studies on tea callus cultivation predominantly emphasize omics research related to secondary metabolite biosynthesis [32], stress defense mechanisms [33], and flavor quality improvement [34], yet there is a lack of systematic analyses regarding the color changes associated with anti-browning treatments. Based on this, our study systematically elucidated the potential molecular mechanisms underlying color changes in tea callus treated with anti-browning agents through a comprehensive comparative analysis of color trait differences, coupled with the integration of transcriptomic and metabolomic profiling.

### 3.1. Differences in Metabolite Accumulation Affecting Callus Color Change

The color differentiation in *C. sinensis* callus is closely linked to the dynamic accumulation of intracellular metabolites, with the differential distribution of phenolic acid compounds and pigment precursor substances being particularly prominent [35]. Studies have demonstrated the substantial expression of flavonoids and phenolic acids in the flesh of *Ipomoea batatas* (sweet potato) with distinct colors [36]. Phenolic acids are crucial for plant growth and development as they facilitate nutrient absorption and enhance photosynthetic efficiency [37]. Moreover, phenolic acids improve plant stress resilience by accumulating under biotic and abiotic stresses to exhibit potent antioxidant activity [38,39]. In this study, we employed targeted phenolic acid metabolomics to elucidate the mechanism underlying color changes in *C. sinensis* callus treated with anti-browning agents and integrated transcriptomics to further dissect the underlying molecular mechanisms. Metabolomic analysis identified 14 phenolic acid metabolites in differently colored callus tissues. These metabolites displayed significantly distinct accumulation patterns across the color groups. Notably, the content of metabolites such as epicatechin, catechin, 4-hydroxybenzoic acid, p-coumaric acid, and protocatechuic acid was significantly higher in red callus tissues compared to in white and green callus tissues. Epicatechin and catechin function as antioxidants during flavonoid biosynthesis, stabilizing both intermediate compounds and final products in the pathway to prevent oxidative degradation [40]. As precursor compounds or key intermediates in the flavonoid metabolic pathway, 4-hydroxybenzoic acid, p-coumaric acid, and protocatechuic acid ensure a continuous and stable supply of substrates for flavonoid synthesis [41]. Through their combined roles in antioxidant protection and enzyme activity regulation, these metabolites not only influence anthocyanin accumulation, but also promote the synthesis of red pigments, thereby contributing to the red coloration of the callus. In contrast, the levels of trans-ferulic acid and vanillic acid in white callus were significantly higher compared to those in red and green callus. In green callus, only the level of salicylic acid was higher than in white callus, whereas the levels of other metabolites were markedly downregulated. This highlights the pivotal role of these metabolites in color regulation. Red callus was enriched with a broader spectrum of metabolites, predominantly intermediates or precursors within the phenylpropanoid metabolic pathway. The downregulation of these metabolites inhibited phenylpropanoid biosynthesis, thereby redirecting carbon sources and energy toward the chlorophyll biosynthesis pathway [39]. This shift promoted chlorophyll accumulation, resulting in efficient chlorophyll deposition and the induction of green pigmentation in callus. Taken together, these findings demonstrate a strong correlation between callus color variation and the accumulation of phenolic acid compounds.

### 3.2. Anti-Browning Agent-Activated Carbon Maintains Green Phenotypes via Enhanced Photosynthesis

Activated carbon (AC) demonstrates remarkable adsorptive capacity, effectively sequestering phenolic substances and oxidation products in the culture medium. Under Pb-contaminated conditions, AC significantly enhances chlorophyll a content, thereby reflecting its protective and restorative effects on the photosynthetic system [42]. This protective effect is likely to be closely linked to the regulation of key gene families in the photosynthetic system. Notably, the *psa* and *psb* gene families, as core regulators of photosynthesis, play an essential role in maintaining chloroplast structure, stabilizing photosynthetic complexes, and encoding critical components of photosystem II, thereby ensuring efficient chlorophyll light capture and the manifestation of plant green phenotypes [43,44]. Transcriptomic analysis showed that in AC-treated callus, genes associated with the *psa* family of Photosystem I (PS I), the *psb* family of Photosystem II (PS II), and the cytochrome b6/f complex, as well as the 1 *petE*, 1 *petF*, and 1 *petC* DEGs involved in photosynthetic electron transport were significantly upregulated. This indicates that the callus might sustain electron flow via both linear and cyclic electron transport pathways, thereby promoting ATP synthesis to maintain chloroplast structure stability and facilitate continuous chlorophyll accumulation. In the F-type ATPase complex, the majority of genes were significantly downregulated. Since callus tissues primarily depend on gluconeogenesis rather than photophosphorylation for energy production, the suppression of ATPase expression minimizes unnecessary energy expenditure, thus promoting reliance on cyclic electron transport. This strategic reallocation of energy resources not only sustains the stability of the photosynthetic system, but also enhances chlorophyll accumulation and reinforces the compactness and stability of cellular structures. Consequently, this leads to the development of a green, dense, and clumped callus morphology [45,46,47]. Metabolomic analysis revealed that the levels of several metabolites in the phenylpropanoid pathway, including trans-cinnamic acid and p-coumaric acid, were significantly reduced in green callus compared to in white and red callus. The downregulation of phenylpropanoid pathway metabolites indicates that the anti-browning agent AC may redirect carbon allocation by preferentially channeling metabolic precursors, such as 4-coumaroyl-CoA, into the chlorophyll biosynthesis pathway. This reduces the competitive consumption of carbon skeletons for lignin and flavonoid biosynthesis, thereby further suppressing the expression of downstream anthocyanin biosynthesis genes. This metabolic and transcriptional cascade regulation established a dynamic equilibrium among “biased carbon allocation, enhanced photosynthesis, and biased pigment biosynthesis,” allowing the AC-treated callus to maintain a stable green phenotype under anti-browning treatment. In summary, AC significantly upregulates the expression of genes belonging to the *psa* and *psb* families in the photosynthetic system, as well as the 1 *petE*, 1 *petF* and 1 *petC* differential genes involved in the photosynthetic electron transport chain by modulating carbon source allocation. This upregulation facilitates chlorophyll accumulation, thereby enhancing photosynthetic efficiency. Additionally, it contributes to maintaining a compact cellular structure, resulting in the formation of a dense, green, and clumped morphology.

### 3.3. Ascorbic Acid Modulates Pigment Formation in Tea Callus by Regulating Key Gene Expression

As a potent antioxidant, ascorbic acid (VC) plays a crucial role in inhibiting oxidative stress in plant tissue culture by scavenging reactive oxygen species (ROS), regulating phenolic metabolism balance, and consequently modulating callus phenotypes [48]. Studies on apple callus have demonstrated that the overexpression of genes associated with ascorbic acid biosynthesis (e.g., *MdCPCL*) results in substantial increases in the ascorbic acid and anthocyanin content, thereby inducing red-colored callus formation [49]. This study further reveals that the exogenous application of ascorbic acid (VC) effectively suppresses browning in *C. sinensis* callus, while promoting the significant reddening of the tissues *CHS* and *CHI*, which are essential synthases in the anthocyanin biosynthesis pathway, and their expression levels directly influence plant coloration [50]. Extensive research has demonstrated that genes such as *CYP75B1*, *C4H*, *F3H*, and *DFR* encode key enzymes in the flavonoid biosynthesis pathway. The expression of these genes regulates the synthesis of anthocyanins and proanthocyanidins. Anthocyanins are critical pigments that determine vibrant colors, including red, purple, and blue, in plants, and play a pivotal role in plant pigment biosynthesis [51,52,53,54]. In this study, the 3 *CHS*, 2 *CHI*, 2 *F3H*, 1 C4H, 2 *DFR*, 1 *ANS*, 3 *ANR*, 1 *ANS*, 1 *FLS*, and 2 *CYP75B1* genes were significantly upregulated in red callus. The correlation analysis between DEGs and DEMs revealed that these genes exhibited a significant positive correlation with 4-hydroxybenzoic acid, p-coumaric acid, and protocatechuic acid. Moreover, 4-hydroxybenzoic acid, p-coumaric acid, and protocatechuic acid function as precursor compounds or critical intermediates in the flavonoid biosynthetic pathway. Their enrichment in VC-treated tissues supplies the necessary substrate for anthocyanin biosynthesis. The upregulation of gene expression, coupled with the accumulation of associated metabolites, modifies the profile of flavonoid metabolites, enhancing anthocyanin synthesis and consequently imparting red pigmentation to the callus. qRT-PCR validation confirmed that the *CHS* and *CYP75B1* genes were specifically expressed in red callus, further suggesting their roles as key regulators underlying the reddening phenotype. In addition, the flavonoid DEGs analysis results indicated that three *ANR genes* were highly expressed in red callus. This redirected a portion of the anthocyanin substrates toward (−)-epicatechin synthesis, and a significant positive correlation was observed between the ANR expression levels and the concentrations of epicatechin and catechin. Notably, the potent antioxidant activity of epicatechin effectively alleviates oxidative browning in plant tissues, thereby preserving color stability by modulating the metabolic balance between anthocyanin and epicatechin [55,56]. Simultaneously, partial genes involved in the photosynthetic pathway were upregulated in red callus, including five upregulated DEGs within the F-type ATP synthase complex. This upregulation facilitates the provision of carbon skeletons and ATP for the flavonoid biosynthesis pathway. However, the subsequent increase in metabolic flux may competitively consume the precursor substances necessary for cell wall polysaccharide synthesis, thereby inducing the remodeling of cell wall components and reducing intercellular adhesion. Consequently, this leads to the loose granular structural characteristics observed in red callus [56]. This study demonstrated that the addition of the anti-browning agent VC promoted flavonoid metabolism in red callus by regulating the expression of key genes (*CHS*, *F3H*, *C4H*, *CYP75B1*, and *ANR*). Specifically, this process not only inhibited oxidative browning, but also enhanced the biosynthesis of phenolic acids within the flavonoid pathway, thereby influencing anthocyanin accumulation and ultimately contributing to the reddening of *C. sinensis* callus.

## 4. Materials and Methods

### 4.1. Experimental Materials

**Figure 10:**
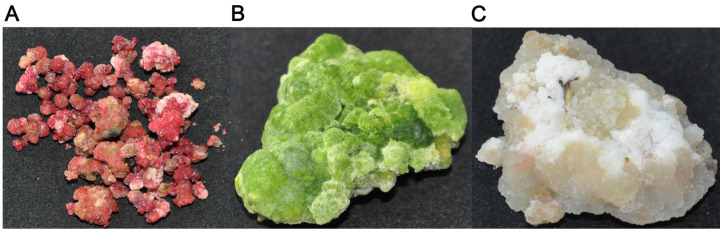
(**A**–**C**) panels, respectively, represent callus tissues treated with ascorbic acid (VC), activated carbon (AC), and polyvinylpyrrolidone (CK).

Young leaves of 2-year-old *C. sinensis* var. *assamica* cv. Mengku, cultivated at the base of the Ancient Tea Tree Research Center, Southwest Forestry University, were utilized as explants for inducing callus formation. To prepare the explants, they were sterilized with 75% ethanol (E809056) for 60 s, followed by rinsing with 1000 mL of sterile water 3–4 times, then sterilized with 5% sodium hypochlorite (76742) for 10 min, and then rinsed with 1000 mL of sterile water 3–4 times. The main leaf veins were removed and cut into 0.5 mm^2^ tissue pieces, which were inoculated in 3.164 g/L B 5 basal medium (PM1321) of 30 g/L sucrose (CS10581) + 6.5 g/Lagar (CA1331) + 2.26 μM 2,4-D (CC3511) + 0.332 μM KT (CK6721), and then light-cultured for 12 h at 25 ± 2 °C to induce and cultivate healing tissues. For the subsequent proliferation and differentiation of healing tissues, the well-grown and uniform healing tissues were selected every 60 ± 2 days for the successor culture. The medium formulation and culture conditions were the same as those of the primary induction system, and the aseptic operation was strictly followed throughout the whole process. The phytohormones used were purchased from Beijing Coolaber Biotechnology Co. After the addition of different anti-browning agents—ascorbic acid (CA2262) 0.04 g/L, activated charcoal (CC3371) 0.04 g/L, and polyvinylpyrrolidone (CP9251) 0.04 g/L—the healing tissues showed three different colors: red, green, and white, and there was a clear difference in the appearance and shape of the white and green healing tissues as dense clumps, and the red healing tissues as lax granules (Figure 10). In this experiment, the healing tissues with the addition of ascorbic acid (VC) and activated charcoal (AC) were used as the treatment group, the healing tissues with the addition of polyvinyl pyrrolidone (CK) were used as the control group, three biological replicates were set up for each experiment, and the collected tissue samples were quickly placed in liquid nitrogen to be frozen and stored at −80 °C for the next assay analysis.

### 4.2. Library Construction and Transcriptome Sequencing

The total RNA of the callus was extracted using TRIzol reagent. RNA samples that passed quality control were selected for high-throughput sequencing, and 0.1 g of each sample was used. Transcriptome sequencing was performed by Shanghai Meiji Biotechnology Co., Ltd. (Shanghai, China). The workflow was as follows: Total RNA was extracted from tissue samples. The concentration and purity of the extracted RNA were measured using a Nanodrop 2000 spectrophotometer (Thermo Scientific, Waltham, MA, USA). The RNA integrity was assessed via agarose gel electrophoresis, and the RQN value was determined using an Agilent 5300 Bioanalyzer (Santa Clara, CA, USA). mRNA was purified from total RNA by binding to magnetic beads coated with Oligo (dT), which selectively hybridizes with the poly (A) tails of mRNA through A-T base pairing. Subsequently, under optimized conditions, the isolated mRNA was fragmented into small pieces of approximately 300 bp using a fragmentation buffer. Using random primers, the first-strand cDNA was synthesized from mRNA templates via reverse transcription. Subsequently, the second-strand cDNA was generated to form a stable double-stranded DNA structure. The cDNA fragments were repaired to blunt ends using the End Repair Mix. Subsequently, a single adenine (A) base was added to the 3′ end of the fragments to facilitate efficient adapter ligation. The adapter-ligated products were then purified and subjected to size selection. Following this, the size-selected fragments underwent PCR amplification to enrich the library. Finally, the library was obtained after purification.

Sequencing was performed on the NovaSeq X Plus platform (Illumina, San Diego, CA, USA) for samples from 9 libraries, using 150 bp paired-end sequencing. The raw sequencing data were subjected to quality control using FastQC (Version 0.0.14) to assess the base quality distribution, GC content, and adapter contamination. Low-quality reads were filtered using fastp (https://github.com/OpenGene/fastp, accessed on 10 October 2024) based on the following parameters: (1) the removal of reads containing adapters; (2) trimming of low-quality bases (quality score < 20) at read ends; (3) exclusion of reads with >10% ambiguous bases (N); and (4) retention of reads ≥ 20 bp after trimming. Sequence comparison of the Clean Reads of each sample was performed with the specified reference genome using HiSat2 (http://ccb.jhu.edu/software/hisat2/index.shtml, accessed on 13 October 2024), reference gene source: *C. sinensis*; reference genome version: shuchazao V2; and reference genome source: (http://tpia.teaplants.cn/download.html, accessed on 13 October 2024). Quality assessment of the comparison results of this transcriptome sequencing was also performed. Differentially expressed genes (DEGs) were identified using DESeq2 (http://bioconductor.org/packages/stats/bioc/DESeq2/, accessed on 14 October 2024), with thresholds of |log2 (fold change)| ≥ 1 and a false discovery rate (FDR) < 0.05. Functional enrichment analysis of DEGs was performed via Goatools (https://github.com/tanghaibao/GOatools, accessed on 14 October 2024) for Gene Ontology (GO) terms and Python SciPy (https://scipy.org/install/, accessed on 15 October 2024) for KEGG pathways, with significance defined as adjusted *p* value < 0.05.

### 4.3. Metabolome Detection

First, 100 mg of the sample was accurately weighed, followed by the addition of a steel ball and 1000 μL of an 80% methanol–water solution (containing 1% vitamin C). The sample was then ground in a cryogenic grinder for 6 min at −10 °C and 50 Hz. Subsequently, ultrasonic treatment was performed at 4 °C for 30 min. After ultrasonic treatment, the sample was centrifuged at 14,000× *g* for 10 min at 4 °C. The supernatant was collected and filtered through a 0.2 μm membrane. Finally, the filtrate was analyzed via LC-ESI-MS/MS (UHPLC-Qtrap) for the qualitative and quantitative detection of target substances in the sample. The metabolomics analysis was conducted by Shanghai Meiji Biotechnology Co., Ltd. Shanghai, China. The screening criteria for differentially expressed metabolites (DEMs) were as follows: metabolites with a fold change (FC) ≥ 1 (indicating a greater than 1-fold difference between the control and experimental groups) and a *p*-value < 0.05 were considered statistically significant. Additionally, metabolites with a Variable Importance in Projection (VIP) score ≥ 1 from multivariate statistical analysis (e.g., OPLS-DA) were further selected as significant differential metabolites. Chromatographic conditions: The analysis was performed on an ExionLC AD system equipped with a Waters BEH C18 liquid chromatography column (100 × 2.1 mm, 1.7 μm). The column temperature was maintained at 35 °C, and the injection volume was set to 1 μL. Mobile phase A consisted of 0.1% formic acid in water, while mobile phase B consisted of 0.1% formic acid in methanol. Mass spectrometry conditions: Analysis was performed using a SCIEX QTRAP 6500+ mass spectrometer, with detection carried out in both positive and negative ionization modes. The following parameters were optimized for the analysis: The Curtain Gas (CUR) was set to 35 arbitrary units, the Collision Gas (CAD) was adjusted to medium, the IonSpray Voltage (IS) was configured to +5500 V in the positive mode and −4500 V in the negative mode, the Temperature (TEM) was maintained at 300 °C, the Ion Source Gas 1 (GS1) was set to 55 arbitrary units, and the Ion Source Gas 2 (GS2) was also set to 55 arbitrary units.

### 4.4. Validation via qRT-PCR

For qRT-PCR validation, ten genes were selected from the list of DEGs. Specifically, five genes were key regulators in flavonoid biosynthesis, and another five genes played crucial roles in the photosynthesis pathway. Additionally, GAPDH was included as an internal reference gene. Primers for the selected DEGs were designed and synthesized by Shanghai Meiji Biotechnology Co., Ltd. The efficiency and specificity of the designed primers were also evaluated.

Quantitative PCR reagents: ChamQ SYBR ColorqPCR MasterMix (2X), Nanjing Novozymes Bio-technology Co. Quantitative PCR instrument: QuantStudio™ 5 384-well fluorescent quantitative PCR instrument, Applied Biosystems, PCR cycling conditions were as follows: 95 °C for 3 min, followed by 95 °C for 5 S. The PCR cycle conditions were as follows, 55 °C 30 S for 40 cycles.

The Ct value difference (ΔCt) between the target gene and the internal reference gene was calculated for each sample. Subsequently, the ΔCt values of the experimental group were compared with those of the control group to determine the ΔΔCt values. Finally, the relative expression levels were calculated using the formula 2(^−ΔΔCt^) [57].

### 4.5. Data Processing

Data processing was performed using Excel (2019), statistical analysis was carried out with SPSS 27.0, and visualizations were created using Origin (2021) and Adobe Photoshop (2023) [58]. A one-way analysis of variance (ANOVA) was utilized to compare the means of the measured parameters. The overall gene expression levels were quantified using RSEM software (Version 1.3.3) [59]. Gene Ontology (GO) enrichment analysis was performed on the genes in the gene set using Goatools [60]. Additionally, KEGG (Kyoto Encyclopedia of Genes and Genome) pathway enrichment analysis for the genes in the gene set was conducted via R software (version 4.4.3) [61].

## 5. Conclusions

This study systematically elucidated the molecular mechanisms underlying the white, green, and red pigmentation of *C. sinensis* callus via integrated transcriptomic and metabolomic analyses. The color phenotypes of the callus were found to be closely linked to the dynamic balance between photosynthesis and flavonoid biosynthesis pathways. Based on the results of the anti-browning agent intervention experiment, it was observed that the addition of VC could regulate the expression of key genes in the flavonoid biosynthesis pathway, thereby inhibiting oxidative browning and promoting the synthesis of the phenolic acid compounds associated with the flavonoid metabolic pathway. This subsequently influences pigment accumulation in the callus tissue, leading to its reddish appearance. However, the high metabolic demands of the callus may trigger the excessive consumption of carbon sources, which ultimately results in the loss of viability and proliferation capacity, causing the callus to exhibit a loose granular morphology. White callus exhibited browning at the late proliferation stage, attributed to insufficient chlorophyll and antioxidant biosynthesis resulting from an inefficient photosynthetic system. This limitation hindered its ability to counteract phenolic oxidation effectively. AC maintains the green phenotype by regulating carbon allocation and upregulating key genes in the *psa*, *psb*, and *pet* families within the photosynthetic system. This not only promotes chlorophyll accumulation, but also enhances photosynthetic efficiency, thereby maintaining cellular structural integrity and ultimately leading to the formation of dense, green callus aggregates. Furthermore, the equilibrium between the antioxidant system and energy metabolism is crucial for sustaining cell division and differentiation capabilities. Consequently, AC demonstrates significant advantages in inhibiting browning, preserving morphological stability, and promoting callus growth. This positions AC as an optimal anti-browning agent with dual functionalities of browning resistance and growth promotion in *C. sinensis* tissue culture systems.

## Data Availability

The original contributions presented in the study are included in the article; further inquiries can be directed to the corresponding author.

## Supplementary Materials

The following supporting information can be downloaded at: https://www.mdpi.com/article/10.3390/plants14101454/s1. Table S1: Sequencing data and alignment results of sequencing data with the reference genome; Table S2: Photosynthesis-related synthetic pathways and functional genes; Table S3: Primers of five selected genes in photosynthesis at differential expression genes for qRT-PCR; Table S4: Flavonoid biosynthesis pathways and functional genes; Table S5: Primers of five selected genes in flavonoid biosynthesis at differential expression genes for qRT-PCR.
